# Spina Bifida

**Published:** 1829-03

**Authors:** 


20. *Spina bifida.*—Having lately heard of a case of this disease in our own city, in which the attending physician opened the sac with a common lancet, and the child died; we are induced to recal the attention of our readers to the most approved method of treating this congenital malady. The following are the remarks of Dr. Blundel, in his lectures at Guy's Hospital:

Infants may be affected at birth with dropsy of the spinal theca, occurring with variety in the anatomical condition of the parts. Sometimes the dropsy is in the theca wholly; sometimes in the theca and the cranium too; and the dropsies may communicate. The spinal marrow may, I believe, be perfect, or the cauda equina may be more or less deficient; the nerves of the lower limbs and pelvis being formed, nevertheless, in all their perfection, and stretching into the cavity of the spine to terminate, as Burns has justly stated, not in the marrow, but in that part of the theca which lines the corresponding arches of the lumbar vertebræ; the nerves, in fact, originating, or rather coalescing, at the theca of the spine. When the arches and spinous processes of the vertebræ are wanting throughout the chain, so that the spinal canal is completely open behind from end to end, the spinal marrow is, I suspect, generally deficient altogether; and, indeed, the disease scarcely belongs to that which I am now considering; but in spina befida generally, there is a deficiency on the back of the lumbar vertebræ only, forming a chasm, at which one or two fingers may be passed down into the cavity of the spine; and above, and perhaps below, to some little extent, the spinous processes separate into two lateral pieces, so as to become forked, whence the disease is frequently denominated spina bifida. "Dum res manent fugiant verba." The appellation is not, perhaps, a good one; but if we understand one another, the terms may pass. Life is short—our time may be laid out on more important matters. Over the lumber chasm, we may find the parts in one or two very different conditions; for sometimes on this part there is a large tumor, bulky as a small orange, covered with a dark rosy red skin, marbled with a leaden livid tint; and, in other cases, we find upon the chasm a circular brown wrinkled scar, broad as a half crown.

and flat. An infant may be born with this tumor lacerated and open. Hydrocephalus, in conjunction with this disease, may become very obvious, in consequence of the enlargement of the cranium, and the widening of the sutures and the fontanels. Mr. Bryant, of Kennington, showed me a fine example of this.

If the medulla spinalis be defective, I presume the case admits of no effectual remedy; but when this is sound, and the disease is, in other respects, favourable, a cure is not impossible; and for this, as for some other useful practical additions to surgery—surely well worth whole volumes of mere musical and well-turned periods—our race is indebted to a man whose name conveys his eulogy,—I mean Sir Astley Cooper. To him, and to my distinguished colleagues, his successors, I must refer you for a fuller exposition of the method of operating; suffice it to remark, that the tumor is punctured with an instrument like a glover's needle, and day after day, by little and little, the fluid is gradually drawn away; the aperture being secured, more or less effectually, after every drawing and pressure being kept up by means of bandage, or otherwise. Forty or fifty times, as I learn, it may be necessary to repeat the punctures; the cyst filling repeatedly, but continually shrinking, till, at length, after a succession of operations, the cyst contracts into a sort of cicatrix lying over the chasm, to be afterwards protected by truss. To open the cyst extensively, and discharge the water at once, is, I believe highly dangerous. In the course of twenty-four hours, death ensued in a case of this kind, narrated to me by one of my pupils. The tumor was mistaken for abscess. The cases with the brown flat scar are are not fit for this operation. In hydrocephalic cases, there is little to be hoped. Infants left to their fate, in this disease, perish after different intervals. They may live for weeks, months, or years. They may even reach to man's estate, always laboring under the disease. If the marrow be defective, the lower parts of the body may be defective in feeling. Dr. Haighton used to relate the case of a boy, who would thrust pins into the skin with little suffering. Acupuncturation sometimes occasions little pain, even in the healthy.

